# Quality and Agronomic Trait Analyses of Pyramids Composed of Wheat Genes *NGli-D2*, *Sec-1^s^* and *1Dx5+1Dy10*

**DOI:** 10.3390/ijms24119253

**Published:** 2023-05-25

**Authors:** Zhimu Bu, Gongyan Fang, Haixia Yu, Dewei Kong, Yanbing Huo, Xinyu Ma, Hui Chong, Xin Guan, Daxin Liu, Kexin Fan, Min Yan, Wujun Ma, Jiansheng Chen

**Affiliations:** 1 State Key Laboratory of Crop Biology/Key Laboratory of Crop Water Physiology and Drought-Tolerance Germplasm Improvement, Ministry of Agriculture/Group of Wheat Quality Breeding, Shandong Agricultural University, Tai’an 271018, China; zhimubu@163.com (Z.B.); m17686274077@163.com (G.F.); yuhaixia66@163.com (H.Y.); 13793022203@163.com (D.K.); m18854801061_1@163.com (Y.H.); mxyv1998@163.com (X.M.); ch18263898681@163.com (H.C.); xinner94@163.com (X.G.); 15053810615@163.com (D.L.); 18554211273@163.com (K.F.); 13653305680@139.com (M.Y.); 2 College of Agronomy, Qingdao Agricultural University, Chengyang District, Qingdao 266109, China

**Keywords:** wheat quality, gene pyramid, agronomic trait

## Abstract

Due to rising living standards, it is important to improve wheat’s quality traits by adjusting its storage protein genes. The introduction or locus deletion of high molecular weight subunits could provide new options for improving wheat quality and food safety. In this study, digenic and trigenic wheat lines were identified, in which the 1Dx5+1Dy10 subunit, and *NGli-D2* and *Sec-1^s^* genes were successfully polymerized to determine the role of gene pyramiding in wheat quality. In addition, the effects of ω-rye alkaloids during 1BL/1RS translocation on quality were eliminated by introducing and utilizing 1Dx5+1Dy10 subunits through gene pyramiding. Additionally, the content of alcohol-soluble proteins was reduced, the Glu/Gli ratio was increased and high-quality wheat lines were obtained. The sedimentation values and mixograph parameters of the gene pyramids under different genetic backgrounds were significantly increased. Among all the pyramids, the trigenic lines in Zhengmai 7698, which was the genetic background, had the highest sedimentation value. The mixograph parameters of the midline peak time (MPT), midline peak value (MPV), midline peak width (MPW), curve tail value (CTV), curve tail width (CTW), midline value at 8 min (MTxV), midline width at 8 min (MTxW) and midline integral at 8 min (MTxI) of the gene pyramids were markedly enhanced, especially in the trigenic lines. Therefore, the pyramiding processes of the *1Dx5+1Dy10*, *Sec-1^S^* and *NGli-D2* genes improved dough elasticity. The overall protein composition of the modified gene pyramids was better than that of the wild type. The Glu/Gli ratios of the type I digenic line and trigenic lines containing the *NGli-D2* locus were higher than that of the type II digenic line without the *NGli-D2* locus. The trigenic lines with Hengguan 35 as the genetic background had the highest Glu/Gli ratio among the specimens. The unextractable polymeric protein (UPP%) and Glu/Gli ratios of the type II digenic line and trigenic lines were significantly higher than those of the wild type. The UPP% of the type II digenic line was higher than that of the trigenic lines, while the Glu/Gli ratio was slightly lower than that of the trigenic lines. In addition, the celiac disease (CD) epitopes’ level of the gene pyramids significantly decreased. The strategy and information reported in this study could be very useful for improving wheat processing quality and reducing wheat CD epitopes.

## 1. Introduction

Wheat is an important staple food crop worldwide. Increasing wheat grain yield and protein content are two important goals for addressing population growth and dietary needs. Wheat is an important strategic material that supplies most of the global grain reserves. Due to rising living standards, consumers and processing enterprises have increased the requirements for wheat quality and food safety. Gene pyramiding refers to the accumulation of beneficial target genes that are dispersed in different genetic resources in the same germplasm to cultivate unique wheat varieties with multiple desired traits [1]. The traditional phenotypic identification method cannot readily be used to efficiently select individual plants that aggregate several target genes. Therefore, the use of molecular-marker-assisted selection is an effective technique for rapidly and accurately identifying lines containing multiple beneficial genes [2].

Crude protein content, protein components and protein component ratios ultimately determine flour strength, applicability and end-use quality [3,4]. Wheat proteins are divided into albumin, globulin, gliadin and glutenin according to their solubilities. Wheat storage protein is composed of gliadin and glutenin, which account for approximately 80% of the total protein content of wheat [5]. The composition and proportion of wheat storage protein affect the wheat gluten quality and dough viscoelasticity; therefore, the different processing characteristics, compositions and proportions of gliadin and glutenin play decisive roles in evaluating wheat [6,7,8,9,10,11]. Scholars have shown that the absolute contents and proportions of storage protein components, such as the glutenin subunit content, insoluble glutenin macromer content and ratio of gliadin to glutenin subunit content, directly affect the processing quality of wheat [12,13,14]. Regarding dough, gliadin mainly determines the adhesion and extensibility, while glutenin mainly determines the elasticity [15]. Glutenin is a multimeric protein that accounts for approximately one tenth of the total protein content of wheat. Glutenin contains many disulfide bonds between and within molecules that can crosslink protein subunits [16]. Glutenin can be divided into a high molecular weight glutenin subunit (HMW-GS) and a low molecular weight glutenin subunit (LMW-GS) [17,18]. Based on their different solubilities in SDS buffer, these subunits can be divided into extractable polymeric protein (EPP) and unextractable polymeric protein (UPP). An important factor determining the quality of wheat is the glutenin content, especially the content of HMW-GS, which greatly impacts wheat quality; specific subunits can significantly improve wheat quality, such as the 1Dx5+1Dy10 subunits. *Glu-A1*, *Glu-B1* and *Glu-D1* loci are located on the long arm of chromosome 1 of wheat. These loci control HMW-GS and significantly affect wheat baking quality [19,20,21,22,23,24]. Moreover, some specific subunits of HMW-GSs significantly contribute to improvements in wheat flour quality [25]. Among the subunits, the 1Dx5+1Dy10 subunit encoded by the *Glu-D1* locus on chromosome 1D contributes the most to the baking quality, and the quality differences between the 1Dx5+1Dy10 subunit and its allelic variants—the 1Dx2+1Dy12 subunit and 1Dx3+1Dy12 subunit—are highly influential [26,27].

Wheat gliadin can be divided into four types—α, β, γ and ω—accounting for 25%, 30%, 30% and 15% of the total amount, respectively [28]. The *Gli-A2*, *Gli-B2* and *Gli-D2* loci located at the *Gli-2* locus on the arm of the partially homologous chromosome 6D encode all α-gliadin, most β-gliadin and some γ-gliadin [29]. Previous studies have shown that gliadin is the most toxic wheat protein component, and it is associated with celiac disease (CD); conversely, glutenin is classified as non-toxic or weakly toxic [30,31,32]. CD, also known as gluten-sensitive enteropathy, is a T-cell-mediated autoimmune disease that results from the ingestion of gluten from certain grains, such as wheat, barley, rye and their derivatives, by susceptible genes [33]. To reduce the toxicity of wheat gluten, several flour treatments (chemical, physical and enzymatic) [34,35,36,37] and genetic approaches (knocking out or silencing gliadin-coding genes) have been developed. RNAi can result in a 60–80% reduction in total gliadin content in bran [38]. However, some negative effects on processing quality have been observed in RNAi wheat lines [38,39]. CRISPR–Cas9 has been used to silence the α-gliadin gene to reduce the immune response by 85%; however, the gluten content is reduced by 85%, thus significantly decreasing processing quality [40]. To date, the greatest challenge has been to find a technological solution for reducing wheat gliadin and increasing gluten protein content while ensuring high yields and a high total protein content. Wang et al. created mutants for the six alcohol-soluble protein chromosome sets in common wheat and found that deleting the *Gli-D2* locus (on chromosome 6D) is beneficial for improving bread processing quality, increasing the lysine contents of seeds and reducing celiac-disease-inducing factors [41]. The deletion of the *G1i-D2* locus reduces the total accumulation of alcohol-soluble proteins, indirectly increases the gluten content, increases the gluten-to-alcohol-soluble protein ratio, and enhances protein disulfide bond isomerase activity, thus increasing the gluten macromer content and the bread processing quality.

The *1BL/1RS* translocation line refers to the wheat–rye translocation line formed by replacing the short arm of chromosome *1B* of wheat with the short arm of chromosome 1R of rye. The derived cultivar can usually retain the beneficial genes from rye and increase the disease resistance capability and high, stable yield potential of wheat [42]. To date, *1BL/1RS* translocation has been widely used in wheat cultivation applications worldwide. However, 1BL/1RS translocation has some negative effects on wheat quality. Many scholars have noted that 1BL/1RS translocation significantly changes storage protein components in terms of quantity and quality, thus weakening gluten strength, creating sticky dough, reducing flour yield and reducing processing quality; the processing quality is determined by various parameters, such as sedimentation value, dough development time and maximum tensile resistance [43,44,45]. These quality defects are mainly caused by the losses in the low-molecular-weight glutenin, γ-gliadin and ω-gliadin gene loci in wheat 1BS or by replacement with rye [46]. Bread quality deterioration is mainly caused by the secalin encoded by 1RS rather than losses in gene loci [47]. The 1RS arm in wheat has end-use quality defects that are partially attributable to the presence of ω-secalins, which are encoded by genes at the *Sec-1* locus [48].

To date, most studies on gene pyramiding have focused on improving the insect and disease resistance rather than wheat quality. *NGli-D2*, *Sec-1^s^* and *1Dx5+1Dy10* have been shown to be important for improving wheat quality. However, the effects of three-gene pyramiding on wheat quality and its main/important agronomic traits have not been studied. In this paper, by using gene pyramids with Zhengmai 366, Zhengmai 7698 and Hengguan 35 genetic background materials, the types of pyramids are identified by molecular-marker-assisted selection; the digenic and trigenic wheat lines that successfully polymerized the 1Dx5+1Dy10 subunit and *NGli-D2* and *Sec-1^s^* genes are identified. The methods by which gene pyramiding affects quality are determined. Through gene pyramiding, the 1Dx5+1Dy10 subunit is introduced, the deterioration effect of ω-secalin in 1BL/1RS translocation on quality is eliminated and the Glu/Gli ratio is increased to obtain high-quality wheat lines. The results of this study provide a theoretical basis for improving the processing quality of wheat flour, and they lay a foundation for building a new wheat quality improvement method based on gene pyramiding.

## 2. Results

### 2.1. Identification and Analysis of Wheat Gene Pyramids

**Figure 1:**
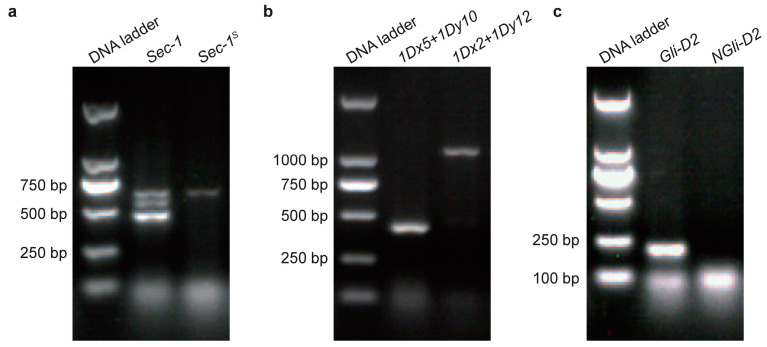
Identification of pyramids of Zhengmai 7698. (**a**) PCR amplification of the *Sec-1^S^* gene. (**b**) PCR amplification of the *1Dx5+1Dy10* subunit. (**c**) PCR amplification of the *NGli-D2* gene.

The gene pyramids were analysed by polymerase chain reactions (PCRs) with gene-specific primers. Three kinds of pyramids were obtained, including type I digenic lines, type II digenic lines and trigenic lines (Figure 1). The type I digenic lines referred to the wheat plants that were composed of *1Dx5+1Dy10* and *NGli-D2* genes. The type II digenic lines referred to the wheat plants that were composed of *1Dx5+1Dy10* and *Sec-1^S^* genes. The trigenic lines referred to the wheat plants that were composed of *1Dx5+1Dy10*, *NGli-D2* and *Sec-1^S^* genes. With the Hengguan 35 genetic background, 127 plants of the type I digenic lines were successfully polymerized in 140 randomly selected F_8_ generations, with a pyramiding rate of 90.7%. The pyramiding rate of the type II digenic lines was 96.3%, and that of the trigenic lines was 81.7%. With the Zhengmai 7698 genetic background, the pyramiding rates of type II digenic lines and trigenic lines were 92.6% and 84.1%, respectively, during F_8_ generation. There were 216 individual plants that were randomly selected during F_8_ generation with the Zhengmai 366 genetic background, and 179 individual plants of the type I digenic lines were successfully polymerized with a pyramiding rate of 82.9% (Table S1).

### 2.2. Effects of Gene Pyramiding on Sedimentation Value and Gluten in Wheat Flour

A comparison between the sedimentation values and gluten contents of gene pyramids with various genetic backgrounds and the wild type are shown in Table 1. With the Hengguan 35 genetic background, there were remarkable differences in the sedimentation values between the type I digenic lines, type II digenic lines and trigenic lines relative to that of the wild type (*p* < 0.05); these values increased by 46.5%, 73.1% and 62.5%, respectively. Among the pyramids, the type II digenic lines had the highest sedimentation value at 48.67 mL. The wet gluten contents of all pyramid types were significantly higher than that of the wild type, which was the gluten index. With the Zhengmai 7698 genetic background, the sedimentation values of the type II digenic lines and trigenic lines were 54.2% and 74.2% higher than that of the wild type, respectively, at the highest sedimentation value of 54.0 mL. The wet gluten content of the type II digenic lines increased by 23.86% relative to that of the wild type. The gluten index of the trigenic lines reached 88.23. With the Zhengmai 366 genetic background, there were no significant differences in the sedimentation value and wet gluten content between the type I digenic lines and wild type. The reason for this phenomenon could be that the wild type of Zhengmai 366 had already reached a considerably high sedimentation value and wet gluten content of 44.17 mL and 36.87%, respectively. This result indicated that the improvement effects of gene pyramiding on sedimentation value and wet gluten content were significant for the lines with low or moderate values. The gluten index of the type I digenic lines reached 95.36%, which was a large increase.

### 2.3. Effects of Gene Pyramiding on Flour Mixograph Parameters

**Figure 2:**
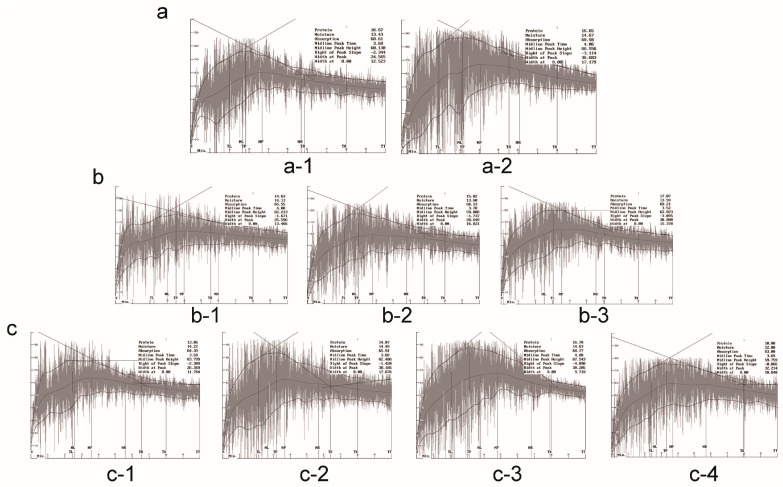
(**a**) Mixograph characteristics of Zhengmai genetic background samples, (**a-1**): CK Zhengmai 366 wild type, (**a-2**): type I digenic lines *(1Dx5+1Dy10* and *NGli-D2*). (**b**) Mixing properties of Zhengmai 7698 genetic background samples, (**b-1**): CK Zhengmai 7698 wild type, (**b-2**): type II digenic lines (*1Dx5+1Dy10* and *Sec-1^S^*) and (**b-3**): trigenic lines (*1Dx5+1Dy10*, *NGli-D2* and *Sec-1^S^*). (**c**) Mixing properties of Hengguan 35, (**c-1**): CK Hengguan 35 wild type, (**c-2**): type I digenic lines (*1Dx5+1Dy10* and *NGli-D2*), (**c-3**): type II digenic lines (*1Dx5+1Dy10* and *Sec-1^S^*) and (**c-4**): trigenic lines (*1Dx5+1Dy10*, *NGli-D2* and *Sec-1^S^*).

With the Hengguan 35 genetic background, the mixograph parameters, midline peak time (MPT), midline peak value (MPV), midline peak width (MPW), curve tail value (CTV) and curve tail width (CTW), of the type I digenic lines, type II digenic lines and trigenic lines were higher than those of the wild type. The mixograph parameters MPT, MPW, CTV and CTW between the type I digenic lines and the wild type were significantly improved, reaching 66.14, 26.7, 51.95 and 9.89, respectively. The MPT (3.86) and MPW (26.44) of the type II digenic lines were significantly higher than those of the wild type. MPT, MPV, MPW, CTV and CTW were significantly improved between the trigenic lines and wild type groups, increasing by 84.6%, 14.5%, 42.4%, 32.3% and 109.5%, respectively. The MPW parameters of the type I digenic lines were higher than those of the other two types of pyramids, the MPT parameters of the type II digenic lines were higher than those of the other two types of pyramids, and the MPV, CTV and CTW parameters of the trigenic lines were higher than those of the other two types of digenic lines (Figure 2c, Table 2). The mixograph parameters midline value at 8 min (MTxV), midline width at 8 min (MTxW) and midline integral at 8 min (MTxI) of the type I digenic lines, type II digenic lines and trigenic lines were higher than those of the wild type. In addition, the mixograph parameters MTxV and MTxW were significantly improved for the type I digenic lines relative to the wild type. MTxV between the type II digenic lines and the wild type significantly increased. MTxV, MTxW and MTxI between the trigenic lines and the wild type significantly improved. The MTxV, MTxW and MTxI parameters of the trigenic lines were higher than those of the other two digenic lines (Figure 2c, Table 2).

With the Zhengmai 7698 genetic background, there were remarkable differences in the mixograph parameters of MPT, MPV, MPW, CTV and CTW between the type II digenic lines and the wild type (*p* < 0.05) and between the trigenic lines and the wild type (*p* < 0.05). For MPV, MPW, CTV and CTW, the trigenic lines were the highest, with values of 65.24, 32.27, 54.05, and 13.25, respectively. The MPT and MPV parameters of the type II digenic lines were significantly higher than those of the wild type, while the trigenic lines parameters, except for MPV, were significantly higher than those of the wild type. The four mixograph parameters of the trigenic lines—MPT, MPW, CTV and CTW—were higher than those of the type II digenic lines (Figure 2b, Table 2). The MTxV, MTxW and MTxI values of the type II digenic lines and trigenic lines were higher than those of the wild type. The MTxV and MTxI parameters of the type II digenic lines were significantly higher than those of the wild type. The MTxV, MTxW and MTxI parameters of the trigenic lines were significantly higher than those of the wild type. The three mixograph parameters of the trigenic lines—MTxV, MTxW and MTxI—were higher than those of the type II digenic lines (Figure 2b, Table 2).

With the Zhengmai 366 genetic background, the mixograph parameters MPT, MPV, MPW, CTV and CTW of the type I digenic lines were higher than those of the wild types. The MPV of the type I digenic lines reached 66.1. The MPW and CTW for the type I digenic lines were 47.6% and 74.2% higher than those of the wild type, respectively. (Figure 2a, Table 2). The mixograph parameters of MTxV, MTxW and MTxI of the type I digenic lines were higher than those of the wild type (Figure 2a, Table 2).

These results indicate that the pyramiding of the *1Dx5+1Dy10*, *Sec-1^S^* and *NGI-D2* genes improved the mixograph characteristics, gluten elasticity, gluten strength and dough resistance of wheat, and it positively affected wheat food processing quality parameters.

### 2.4. Effects of Gene Pyramiding on Grain Protein Components

**Figure 3:**
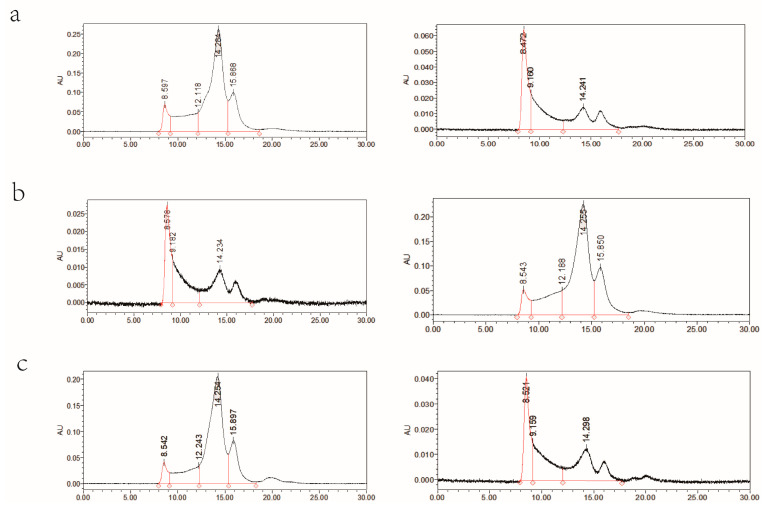
(**a**) Size exclusion (SE)-HPLC chromatogram of Zhengmai 366; (**b**) SE-HPLC chromatogram of Hengguan 35; and (**c**) SE-HPLC chromatogram of Zhengmai 7698.

The protein components of different gene pyramids and wild types were separated by high-performance liquid chromatography (HPLC). The peak areas of different components were calculated to obtain the UPP% [UPP/(UPP+EPP)] and Glu/Gli ratio. The overall performance of the gene pyramid protein components was better than that of the wild type. With the Hengguan 35 genetic background, the UPP% of the type I digenic lines reached 0.38, which was higher than that of the wild type, and the Glu/Gli ratio was significantly higher than that of the wild type by 36.4%. The Glu/Gli ratio of the type II digenic lines (0.54) was higher than that of the wild type (0.44), and the UPP% of the type II digenic lines was significantly higher than that of the wild type, with 0.48 as the highest of the four pyramids. The UPP% and Glu/Gli ratio of the trigenic lines were significantly higher than those of the wild type. The UPP% of the type II digenic lines was higher than that of the trigenic lines by 9.1% and significantly higher than that of the type I digenic lines by 15.8% (Figure 3b, Table 3). With the Zhengmai 366 genetic background, the Glu/Gli ratio of the type I digenic lines was significantly higher than that of the wild type, but the UPP% was not higher than that of the wild type (Glu/Gli ratios of 0.64 vs. 0.51 and UPP% values of 0.40 vs. 0.41) (Figure 3a, Table 3). The UPP% and Glu/Gli ratio of the type II digenic lines and trigenic lines were significantly higher than those of the wild type under the genetic background of Zhengmai 7698, while the type II digenic lines had the highest UPP% of 0.44. The UPP% of the type II digenic lines in this genetic context was higher than that of the trigenic lines, while the Glu/Gli ratio was slightly lower than that of the trigenic lines (Figure 3c, Table 3). Based on the above six pyramids from different genetic backgrounds, the Glu/Gli ratio of the type I digenic lines and trigenic lines containing the *NGli-D2* locus was higher than that of the type II digenic lines without the *NGli-D2* locus. The trigenic lines with Hengguan 35 as the genetic background had the highest Glu/Gli ratio.

### 2.5. Determination and Analysis of CD Epitopes in Near-Isodeletion Lines at the NGli-D2 Locus

**Figure 4:**
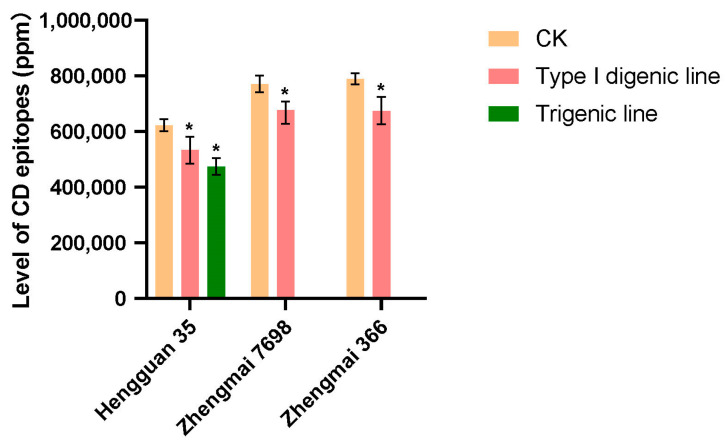
Comparison of CD epitopes between wild type and pyramids. The asterisk in the figure indicates significant differences between wild type and pyramid according to Tukey’s test (*p* < 0.05). CK: wild type; type I digenic lines: *1Dx5*+*1Dy10* and *NGli-D2* pyramids; and trigenic lines: *1Dx5*+*1Dy10*, *NGli-D2* and *Sec-1^S^* pyramids.

To verify whether eliminating the *Gli-D2* locus could reduce the CD epitope, a G12 antibody was used for an enzyme-linked immunosorbent assay (ELISA). With the Hengguan 35 genetic background, there were significant differences between the type I digenic lines, trigenic lines and wild type. The CD epitopes of the type I digenic lines and trigenic lines were significantly lower than those of the wild type. With the Zhengmai 7698 genetic background, there was a significant difference between the trigenic lines and the wild type, and the CD epitope of the trigenic lines was significantly lower than that of the wild type. With the Zhengmai 366 genetic background, there was a significant difference between the type I digenic lines and the wild type, and the CD epitope of the type I digenic lines was significantly lower than that of the wild type (Figure 4). There was a very significant positive correlation between gliadin and CD epitopes, and the correlation coefficient was r = 0.89; there was no correlation between the Glu/Gli ratio and CD epitopes.

### 2.6. Effects of Gene Pyramiding on Agronomic Traits

In the genetic background of Zhengmai 366, compared with the wild type, the type I digenic lines showed a significant difference in the thousand-kernel weight, whereas there were no significant differences in the spike length, number of spikelets, number of grains per panicle or plant height. With the Zhengmai 7698 genetic background, the spike length, spikelet number, grains per spike, plant height and thousand-kernel weight values of the type II digenic lines and trigenic lines were higher than those of the wild type except for plant height; however, there were no significant differences compared to the wild type. The spike length and spikelet number values of the type II digenic lines and trigenic lines were relatively consistent, but the plant height and thousand-kernel weight values of the type II digenic lines were higher than those of the trigenic lines; however, the number of grains per spike was slightly lower than that of the trigenic lines. With the Hengguan 35 genetic background, there were no significant differences in spike length, spikelet number and grains per panicle between the type I digenic lines, type II digenic lines and trigenic lines. The plant height traits of the three types of pyramids were lower than those of the wild type, while those of the type II digenic lines were significantly lower than those of the wild type. The thousand-kernel weight values were higher in the three types of pyramids than in the wild type, while those of the type II digenic lines were significantly higher than those of the wild type. In conclusion, the agronomic traits of the three types of pyramids experienced no significant changes relative to those of the wild type; additionally, there were no negative impacts on the agronomic traits due to gene pyramiding (Table 4).

## 3. Discussion

### 3.1. Marker-Assisted Selection Accelerating the Cultivation of High-Quality Wheat through Gene Pyramiding

Exploitation, research and utilization genes controlling wheat quality, agronomic traits and resistance are highlighted in wheat cultivation programs. It is critical to determine an approach for aggregating desirable genes in the same germplasms and lines, and molecular marker technology plays an important role in multiple gene pyramiding during cultivation. This technology can be used throughout the growth of plants without disease, especially to shorten the cultivation time through rapid and accurate identification of target genes [49]. To date, it has been time-consuming to obtain a high yield, stress resistance and quality simultaneously through conventional cultivation methods. Therefore, a combination of molecular marker technology and conventional cultivation techniques, in addition to the introduction of specific PCR marker technology, to select high-quality and high-yield offspring have been verified to accelerate cultivation [50]. To date, most studies of gene pyramids have focused on disease resistance and yield, while gene-pyramiding-related quality is still poor [1]. In this study, three wheat cultivars—Hengguan 35, Zhengmai 366 and Zhengmai 7698—were used to improve wheat quality by polymerizing three high-quality beneficial genes through molecular-marker-assisted selection. Different types of gene pyramids were obtained. This approach was proven to have high application value in terms of subsequent wheat cultivation progress. The results of this study showed that under the three types of genetic backgrounds, gene pyramids positively affected quality characteristics, mixograph parameters and protein components relative to the wild type; these results are generally consistent with those of previous studies [41].

### 3.2. Improvement in Gene Pyramiding Affected by Genetic Background and Complementarity Factors

Studies have shown that substituting the *1Dx2+1Dy12* subunit with *1Dx5+1Dy10* could improve the quality of gluten, including the good manufacturing practices (GMPs); however, the dough applicability was negatively affected, and the improvement range was affected by the genetic background [51]. In this study, different pyramids resulted in different changes in quality indicators. The reason for this result could be the effects of combinations of different gene pyramids and the genetic backgrounds of materials. The results of this study showed that under the three genetic backgrounds, gene pyramids positively affected quality characteristics, such as the mixing properties, sedimentation value and protein components, and these results were consistent with those of previous studies [41]. The proportion of storage protein components was also affected by genotype, which was consistent with the results of previous studies [52,53]. We detected an improvement in gene pyramiding based on background genetics. Zhengmai 366 and Zhengmai 7698 were used in this study, and they are popular wheat varieties with strong gluten contents. Through gene pyramiding, the gluten strength was further improved, making the varieties more suitable for bread use. Hengguan 35 is a medium gluten wheat, and the type I and type II digenic lines and trigenic lines improved the sedimentation value by 46.5%, 72.6% and 62.5%, respectively, compared with the wild type. Hengguan 35 improved more than the strong gluten varieties Zhengmai 366 and Zhengmai 7698.

It is necessary to consider the degree of complementarity between the target gene and the quality genetic background of recurrent wild types and whether there is interference from other factors, such as the complementarity of protein quality and content. The complementation of glutenin and gliadin components and secalin produced by the 1B/1R translocation line could make the dough sticky, affecting the goal of improving wheat quality [54]. ω-Secalin is the main factor affecting the protein quality of 1BL/1RS wheat [55]. With the 1BL/1RS translocation, the total expression of secalin decreased, and the gluten index, sedimentation value and stability time of transgenic lines significantly increased; however, the agronomic traits were not affected [56].

### 3.3. Significance of 1Dx5+1Dy10, Sec-1^s^ and NGli-D2 Gene Pyramiding for Wheat Processing Quality and Food Safety Improvement

Gene pyramiding affected the proportion of protein components and improved processing quality, thus improving wheat flour gluten strength and kneading resistance and negatively impacting the applicability of dough to a certain extent. This result could be caused by the deletion of some gliadin genes. The deletion of the *Sec-1* locus positively affected farinographic parameters (development time and stability time) and mixograph parameters (MPW and MTxW) [43]. The Glu/Gli ratio was used as an early generation selection indicator of non-translocation lines to improve quality. The strains with better dough rheological properties in non-translocation lines had increased Glu/Gli ratios [57]. Previous research showed that the mixograph parameters of the improved non-translocation system improved to varying degrees, and the defect of a decline in flour processing quality caused by 1BL/1RS improved [58]. Generally, high-quality subunits could contribute positively to flour quality. The 1BL/1RS translocation line had a negative effect on flour quality, while 1Dx5+1Dy10 subunits at the *Glu-D1* locus compensated for the negative impact of 1B/1R translocation on gluten strength [59].

Wheat protein composition is an important factor determining flour quality, especially the composition and quantity of storage protein and the applicability of dough, which are closely related. The 1Dx5+1Dy10 subunit, Glu/Gli ratio and UPP%, were important factors for determining the baking quality of bread [60]; however, not all wheat varieties containing the 1Dx5+1Dy10 subunit had good processing quality stability. Studies have shown that dough containing the 1Dx5+1Dy10 subunit had lower applicability than dough containing the 1Dx2+1Dy12 subunit, but dough containing the 1Dx5+1Dy10 subunit had more SDS-insoluble glutenin and a longer stability time than dough containing the 1Dx2+1Dy12 subunit [61]. The results of this study showed that the sedimentation value and mixograph parameters of the three types of pyramids containing 1Dx5+1Dy10 subunits were higher than those of Hengguan 35 containing 1Dx2+1Dy12 subunits, which was consistent with previous research results [57]. Additionally, pyramids containing *Sec-1^S^* significantly affected quality improvement, which was consistent with previous studies [43].

α-, ω- and γ-gliadins are important immunogens that cause T-cell immune CD, and α-gliadin is the main cause of CD [59,62]. Shewry et al. [63] proposed using transgenic and gene editing methods to reduce the epitopes causing CD without changing the processing quality of wheat. In this study, the CD antigen epitope of wheat was effectively reduced through the deletion of the *Gli-D2* locus; this finding was consistent with those of previous studies [41]. In addition, the correlation between the CD epitope and Glu/Gli ratio was analysed. The results showed that the CD epitope was significantly correlated with gliadin, but there was no correlation with the glutathione ratio. The research results are of great significance for improving food safety.

### 3.4. 1Dx5+1Dy10, Sec-1^s^ and NGli-D2 Gene Pyramiding Have No Negative Impact on Wheat Agronomic Traits

The effects of previous quality improvements on agronomic traits were mostly limited to changes in a single locus, and the results were different for various backgrounds and materials. Wang et al. analysed the agronomic characteristics of 93 high-generation lines of common wheat with different HMW-GS compositions, and they found that changes in HMW-GS composition did not affect agronomic characteristics [64]. Some scholars have combined two translocation lines through hybridization, and identifying and selecting the best translocation line. After investigation, the agronomic characteristics of the two translocation lines did not deteriorate after combination. In contrast, the number of spikelets and thousand-kernel weight increased [65]. Through gene pyramiding, wheat yield component factors, such as thousand-kernel weight and spikelet number, were improved to varying degrees. Therefore, in theory, wheat yield increased, but the specific impact on yield required further data collection and analysis. In total, gene pyramiding did not negatively impact yield traits. The introduction of the 1Dx5+1Dy10 subunit did not affect the agronomic traits of present and future generations of wheat [66]. Multiple high-quality HMW-GS genes of Xiaoyan 22 were introduced into local high-yield lines, and quality parameters, such as stability times of future wheat generations, greatly improved; however, the agronomic traits did not change [66]. Chai et al. used RNA interference technology to cause the non-expression of ω-secalin to improve the processing quality of Jinhe 9132 without adverse effects on agronomic characteristics [56]. This study showed that the ear length, spikelet, grains per ear, plant height and thousand kernel weight values of the gene pyramids were not significantly lower than those of the parent; the agronomic characteristics of some pyramids were slightly higher than those of the wild type. Therefore, *1Dx5+1Dy10, Sec-1^s^ and NGli-D2* gene pyramiding did not negatively impact agronomic traits.

## 4. Materials and Methods

### 4.1. Plant Materials and Cultivation Procedure

Hengguan 35 has the following characteristics: a dwarf stalk, high spike number, drought resistance, water-saving ability and high wheat yield. Zhengmai 7698 has the following characteristics: a weak spring, multiple spikes and strong gluten varieties. Zhengmai 366 has the following characteristics: semi-wintering abilities, multiple spikes and strong gluten wheat varieties. These varieties are well known in China and are planted throughout the country. Hengguan 35 is a winter wheat with the widest adaptation range and the greatest drought and water savings, showing high yields across northern and southern regions of China. The mutants of *Sec-1^s^* and *NGli-D2* obtained by radiation mutagenesis Hengguan 35 and Xiaoyan 81 were provided by the Institute of Genetics and Developmental Biology, Chinese Academy of Sciences. The mutants were further backcrossed to Hengguan 35, Zhengmai 7698 and Zhengmai 366 to obtain BC_3_F_2_. The breeding scheme of these wheat BC_3_F_2_ materials were described previously [5]. The plant materials were planted at the experimental station of Shandong Agricultural University (Tai’an, Shandong) from 2016 to 2021; after 6 generations of continuous self-pollination identification and screening, different BC_3_F_8_ pyramids of *1Dx5+1Dy10*, *Sec-1^s^* and *NGli-D2* were obtained. The method of planting was to select backcross materials that were identified and polymerized, planting was continued after harvest, individual plants were identified and individual plants of the identified screened materials were harvested. Three years of data on BC_3_F_6_ to BC_3_F_8_ pyramids from 2019 to 2021 were used for quality and agronomic trait analyses.

### 4.2. Extraction of Wheat Leaf DNA

The cetyltrimethylammonium bromide (CTAB) extract was preheated in a constant temperature water bath at 65 °C. The fresh leaves were weighed in appropriate amounts and ground to a powder. Then, 700 μL of CTAB extract was added at 65 °C to a centrifuge tube with ground leaves, oscillated until well mixed, and returned to the water bath for 1–2 h. The sample was removed, and an equal volume (700 μL) of chloroform isoamyl alcohol mixture (24:1) was added, mixed thoroughly and left in a −20 °C fridge for 20 min. The centrifuge tube was centrifuged at 12,000 rpm for 10 min, the supernatant was aspirated and 600 μL of cold isopropanol was added, after which the tube was left in the refrigerator at −20 °C for approximately 20 min. After centrifugation at 12,000 rpm for 10 min, the supernatant was discarded, the sample was washed repeatedly with 70% ethanol and the sample was left at room temperature for 5 min. The appropriate amount of ddH_2_O was poured into the sample, which was dissolved, mixed and stored at −20 °C.

### 4.3. Agarose Gel Electrophoresis

For 1% agarose gel configuration, an appropriate amount of tris–acetate–ethylenediaminetetraacetic acid (1 × TAE) was mixed with an appropriate amount of agarose powder, and it was heated until it was completely dissolved. After the mixture temperature decreased to approximately 60 °C, ethidium bromide (EB) dye was added. For glue plate preparation, mixture was poured into a glue-making tank, and a comb was inserted until the gel was completely cooled. For the dot sample, there was an 8 μL DNA sample per dot sample well. For electrophoresis, the electrophoresis apparatus was 120 V, 180 mA, 30 min.

### 4.4. Preparation of SDS—PAGE Solution

For extract A, isopropyl alcohol and distilled water were mixed in a 1:1 ratio at a constant volume of 100 mL. After blending, the buffer solution was dissolved in a water bath at 60 °C and stored at 4 °C. For HMW-GS sample extract, 10 mL of distilled water and 200 mg of dithiothreitol (DTT) were added to 10 mL of mother liquor and dissolved completely, and the sample was stored at 4 °C. The gel storage solution was 30% Acr-0.8% Bis solution. For the gel accumulation buffer (0.5 M Tris-HCl, pH = 6.8), 6.05 g of Tris was dissolved in 40 mL of water, and the pH was adjusted to 6.8 with 1 M HCl at a constant volume of 100 mL. For separation buffer solution (1.5 M Tris-HCl, pH = 8.8), a total of 18.20 g of Tris-HCl was dissolved in 30 mL of water, and the pH was adjusted to 8.8 with 1 M HCl, at a constant volume of 100 mL. For 10% sodium dodecyl sulfate (SDS) solution, 5 g SDS was dissolved in 30 mL of distilled water at a constant volume of 50 mL. For 1.5% ammonium persulphate (AP) solution, 0.15 g of AP was dissolved in a constant volume of 10 mL and stored at 4 °C. For electrode buffer, 14.41 g Gly, 3.0 g Tris and 1.0 g SDS were dissolved; the pH was adjusted to 8.3 at a constant volume of 1000 mL, and the buffer was set at room temperature.

### 4.5. Protein Extraction from Wheat Grains

Whole wheat flour (0.03 g) was weighed. Next, 300 μL of extract A was added, shaken and incubated at 65 °C for 30 min; the sample was shaken twice during soaking. Centrifugation was performed for 10 min at 10,000 rpm and 20 °C, the supernatant was discarded and 210 μL of HMW-GS sample extract was added; the mixture was mixed by shaking, and it was heated at 60 °C for 2 h. During heating, the mixture was shaken 3 times. After centrifugation for 10 min at 10,000 rpm and 20 °C, 100 μL of supernatant was collected and stored in a refrigerator at 4 °C.

### 4.6. SDS—PAGE Agarose Gel Electrophoresis

For gel preparation, agar powder (1.0 g) was weighed in 100 mL of electrode buffer and heated until it was completely dissolved, and a gel back cover was used. For sampling and electrophoresis, 10 μL of sample solution was placed in each tank, and the electrophoresis apparatus was set with a current of 15 mA, voltage of 100 V, power of 50 W and time of 9 h. For dyeing, the separation gel and dye were maintained. Decolorization was conducted with distilled water. For qualitative analysis, the HMW-GS type of the tested strain was determined according to the electrophoretic band of the control.

### 4.7. Wheat Flour Milling and Determination of Protein Content and Moisture

After removing impurities and moistening the wheat, flour was ground using a hammer-type experimental mill (Dacheng Photoelectric Instrument Co., Hangzhou, China). The protein content and moisture of the flour were determined by a DA7200 near-infrared (NIR) instrument (Perten, Stockholm, Switzerland).

### 4.8. Determination of Wheat Flour Sedimentation Value

The sedimentation value of wheat (Zeleny method) was determined according to the AACC56-61A method using LYZY-2 sedimentation value tester (Liang Yuan Analytical Instruments Co., Zhengzhou, China). Diluted lactic acid was refluxed for 6 h to obtain the lactic acid solution. Bromophenol blue (0.004 g) was weighed and diluted to 1000 mL with water, to obtain a bromophenol blue solution. The lactic acid mixture was obtained by mixing 180 mL of lactic acid solution and 200 mL of isopropanol, by adding distilled water to 1000 mL and by letting the mixture stand for 48 h. Then, 3.2 g of flour was weighed. Next, 50 mL of bromophenol blue was added, and the sample was allowed to stand for 5 min. Then, the sample was shaken left and right with both hands to evenly suspend the flour in solution. The tube was loaded into a shaker and placed in a slot. When the time was complete, the tube was removed, and 25 mL of lactic acid mixture was immediately added. Then, the tube was placed on a shaker for 5 min. The volume of the precipitate was read after 5 min of standing, which was the sedimentation value. The results were expressed for 3.2 g of flour with a moisture content of 14% on a wet base. If the moisture content of the flour sample was not 14%, the uncorrected sedimentation value was converted to the sedimentation value of the 14% wet basis according to the following formula:

$$\mathrm{Water}\;\mathrm{content}\;14\%\;\mathrm{wet}\;\mathrm{basis}\;\mathrm{sedimentation}\;\mathrm{value}\;\;\;\;\;\;\;\;\;\;\;\;\;\;\;\;\;\;\;\;\;\;\;\;\;\;\;\;\;\;\;\;\;\;\;\;\;\;\;\;\;\;\;\;\;\;\;\;\;\;\;\;\;\;\;\;\;\;\;\;\;\;\;\;\;\;\;\;\;\;\;\;\;\;\;\;\;\;\;\;\;\;\;=\mathrm{uncorrected}\;\mathrm{sedimentation}\;\mathrm{value}\times \frac{100-14}{100-\mathrm{flour}\;\mathrm{water}\;\mathrm{content}}$$

### 4.9. Determination of Wheat Mixograph Parameters

Mixograph parameters: Mixograph parameters were analysed using a mixograph according to the American Association of Cereal Chemists (AACC) method 54-40. According to the calculation formula, the correct amount of sample was weighed in a mixing bowl, the flour was gently pushed in the bowl with a tongue-shaped block to form a triangular cavity, and an accurate amount of purified or distilled water was added to the cavity. The stirring time was set to 10 min for all samples. The computer automatically saved the results at the end of the experiment. Sample weighing, water absorption and water addition were defined by the following equation:

$$\mathrm{Sample}\;\mathrm{weighing}\left(g\right)=10\times \frac{0.86}{1-\mathrm{actual}\;\mathrm{water}\;\mathrm{content}}$$

$$\mathrm{Water}\;\mathrm{absorption}\left(g\right)=\left(\mathrm{protein}\;\mathrm{content}\times 1.5\right)+43.6$$

$$\mathrm{Water}\;\mathrm{addition}\left(g\right)=10-\mathrm{sample}\;\mathrm{weight}+0.1\times \mathrm{water}\;\mathrm{absorption}$$

### 4.10. Determination of Wheat Protein Fractions

HPLC was used to separate the alcohol-soluble protein, albumin, globulin, SDS-soluble glutenin and SDS-insoluble glutenin. The chromatographic system involved a Waters 515 chromatograph and a Waters 2478 detector, and the workstation software was Empower. The sample injection volume of the sample ring was 60 μL, and the sample ring volume was 20 μL. The column was TSK G4000 SW (7.5 mm × 300 mm), and the guard column was TSK G3000SW (7.5 mm × 75 mm). For extraction solution configuration, 0.1 mol/L sodium phosphate buffer solution (pH = 6.9) + 2% (*w*/*v*) was used. For SDS buffer configuration, 0.1 mol/L sodium phosphate buffer solution (pH = 6.9) + 0.1% (*w*/*v*) SDS was used.

To extract albumin, globulin, alcohol-soluble protein and SDS-soluble glutenin, 0.01 g of whole wheat flour was weighed on a 10,000 ppm balance. One milliliter of extraction solution was added to whole wheat flour, and the sample was shaken at 60 °C in a constant temperature shaker for 2 h and centrifuged at 12,500× *g* and 20 °C for 30 min. The supernatant was collected and filtered through a membrane. To extract clear protein, globulin and SDS-insoluble glutenin, 1 mL of extraction solution was added to the residue, sonicated with an output 20 W and frequency of 23 kHz for 15 s; then, it was vortexed for 2 min, shaken with a thermostatic oscillator for 30 min and centrifuged at 12,000 rpm for 30 min at 20 °C. The membrane was filtered, and the supernatant was collected.

### 4.11. Determination of Agronomic Traits in Wheat

To determine the panicle length, spikelet number and grain number per panicle, ten plants were randomly selected and investigated in the laboratory. For the plant height, wheat was measured in the field at maturity with a ruler. For thousand-kernel weight, after wheat ripening and threshing, 1000 grains of wheat without impurities were randomly selected and weighed, and the average value was taken after three repeated measurements.

### 4.12. Determination of CD Epitope in Wheat Flour

The AgraQuant^®^G12 GlutenELISA Test Kit manufactured by ROMER LABs, Inc. (Beijing, China). was used in this analysis. A representative 0.025 g sample was weighed, 2.5 mL of extract was added and the mixture was shaken violently to prevent clumping. The sample was placed in a 50 °C water bath for 40 min, during which the sample was oscillated 3~4 times. The sample was cooled to room temperature and mixed with 7.5 mL of 80% anhydrous ethanol for 1 h on a vertical mixer. After centrifugation at 12,000 rpm for 10 min at room temperature, the gluten protein solution was collected. An appropriate amount of gluten protein solution was absorbed and diluted with the sample diluent to an appropriate concentration. The diluted sample was added to the enzyme-labelled plate with the appropriate antibody and incubated at room temperature for 20 min. The sample was discarded and washed five times with 250 μL of washing buffer. Then, 100 μL of enzyme-coupled solution was added, and incubation was performed at room temperature for 20 min. The enzyme-coupled solution was poured out and rinsed with 250 μL of washing buffer. Thereafter, 100 μL of substrate solution was added, and incubation was performed at room temperature away from light for 20 min. Then, 100 μL of reaction stop solution was added, and the absorbance was measured immediately after mixing. The corresponding concentration was calculated according to the standard curve.

### 4.13. Data Processing

Excel 2010 was used for data processing, and the differences between the different groups were tested by one-way analysis of variance (ANOVA) (Tukey’s test). All statistical analyses were performed with International Business Machine (IBM) Statistical Product and Service Solutions (SPSS) Statistics 22.0 software (SPSS Inc., http://www.casinovendors.com/vendor/spss-inc/ (accessed on 15 April 2023)).

## Figures and Tables

**Table 1:** Effects of different gene pyramiding processes on sedimentation value, gluten content and gluten index.

Background	Pyramid	Sedimentation Value (mL)	Wet Gluten Content (%)	Gluten Index (%)
Hengguan 35	CK	28.2 ± 3.4b	33.83 ± 0.81b	63.67 ± 2.02b
	Type I digenic lines	41.30 ± 4.88a	38.6 ± 2.48ab	92.58 ± 4.15a
	Type II digenic lines	48.67 ± 6.30a	44.07 ± 7.47a	84.03 ± 14.47a
	Trigenic lines	45.82 ± 6.08a	43.35 ± 6.48ab	82.69 ± 3.52a
Zhengmai 7698	CK	31 ± 1.21b	37.47 ± 3.25b	70.2 ± 1.11a
	Type II digenic lines	47.81 ± 4.85a	46.41 ± 5.23a	76.37 ± 10.04a
	Trigenic lines	54.00 ± 2.88a	42.67 ± 6.96ab	88.23 ± 5.59a
Zhengmai 366	CK	44.17 ± 0.76a	36.87 ± 1.44a	81.37 ± 3.95b
	Type I digenic lines	45.61 ± 4.22a	38.06 ± 1.7a	95.36 ± 1.91a

The different lowercase letters in the table indicate significant differences between wild type and the pyramids according to Tukey’s test (*p* < 0.05). CK: control (wild type of corresponding wheat variety), type I digenic lines: *1Dx5+1Dy10* and *NGli-D2* pyramids; type II digenic lines: *1Dx5+1Dy10* and *Sec-1^S^* pyramids; trigenic lines: *1Dx5+1Dy10*, *NGli-D2* and *Sec-1^S^* pyramids.

**Table 2:** Effects of different gene pyramiding techniques on mixograph parameters.

Background	Pyramids	MPT (Midline Peak Time)	MPV (Midline Peak Value)	MPW (Midline Peak Width)	CTV (Curve Tail Value)	CTW (Curve Tail Width)	MTxV (Time = 8 min Value)	MTxW (Time = 8 min Width)	MTxI (Integral Area)
Hengguan 35	CK	1.82 ± 0.02b	59.71 ± 2.08b	16.36 ± 1.21b	41.12 ± 0.58b	4.81 ± 0.58b	43.77 ± 1.53b	3.93 ± 0.58b	410.54 ± 51.03b
	Type Idigenic lines	3.59 ± 0.72a	66.14 ± 8.18ab	26.70 ± 8.55a	51.95 ± 7.19a	9.89 ± 4.95a	54.43 ± 7.39a	11.24 ± 3.36a	444.86 ± 44.61ab
	Type IIdigenic lines	3.86 ± 0.37a	66.91 ± 7.17ab	26.44 ± 4.80a	48.95 ± 10.47ab	7.12 ± 3.15ab	54.62 ± 6.51a	9.24 ± 3.54ab	453.34 ± 51.00ab
	Trigenic lines	3.36 ± 0.74a	69.91 ± 5.38a	23.30 ± 3.73ab	53.43 ± 4.32a	10.08 ± 2.12a	56.30 ± 4.29a	10.07 ± 3.51a	474.12 ± 33.74a
Zhengmai 7698	CK	1.88 ± 0.15b	56.93 ± 1.15b	21.32 ± 1.00b	41.59 ± 1.53b	3.91 ± 0.25b	40.07 ± 1.15b	4.41 ± 0.26b	387.07 ± 10.00b
	Type IIdigenic lines	3.19 ± 0.70a	66.48 ± 6.42a	20.44 ± 5.04b	48.16 ± 2.98ab	5.66 ± 0.85b	50.39 ± 3.23a	6.33 ± 1.41b	441.25 ± 26.65a
	Trigenic lines	4.23 ± 1.08a	65.24 ± 6.36ab	32.27 ± 7.68a	54.05 ± 7.89a	13.25 ± 3.99a	57.16 ± 8.27a	16.10 ± 4.35a	447.84 ± 39.57a
Zhengmai 366	CK	3.21 ± 0.09b	58.31 ± 1.00b	19.01 ± 2.00b	49.95 ± 2.08a	5.43 ± 0.58b	47.07 ± 2.65a	7.83 ± 0.58b	413.30 ± 10.00a
	Type Idigenic lines	4.09 ± 0.57a	66.10 ± 4.94a	28.05 ± 5.43a	52.32 ± 5.01a	9.46 ± 2.12a	55.54 ± 5.56a	12.48 ± 2.86a	499.78 ± 32.83a

The different lowercase letters in the table indicate significant differences between wild type and the pyramids according to Tukey’s test (*p* < 0.05). CK: control (wild type), type I digenic lines: *1Dx5*+*1Dy10* and *NGli-D2* pyramids, type II digenic lines: *1Dx5*+*1Dy10* and *Sec-1^S^* pyramids, and trigenic lines: *1Dx5*+*1Dy10*, *NGli-D2* and *Sec-1^S^* pyramids.

**Table 3:** Effects of different pyramids on the content of wheat protein components with the same genetic background.

Background	Pyramid	UPP%	Glu/Gli
Hengguan 35	CK	0.24 ± 0.01c	0.44 ± 0.09b
	Type I digenic lines	0.38 ± 0.06bc	0.60 ± 0.65a
	Type II digenic lines	0.48 ± 0.06a	0.54 ± 0.60ab
	Trigenic lines	0.44 ± 0.11ab	0.65 ± 0.13a
Zhengmai 7698	CK	0.21 ± 0.02c	0.44 ± 0.18b
	Type II digenic lines	0.44 ± 0.08a	0.56 ± 0.07a
	Trigenic lines	0.35 ± 0.05b	0.57 ± 0.05a
Zhengmai 366	CK	0.41 ± 0.02a	0.51 ± 0.02b
	Type I digenic lines	0.40 ± 0.06a	0.64 ± 0.05a

The different lowercase letters in the table indicate significant differences between wild type and the pyramids according to Tukey’s test (*p* < 0.05). CK: wild type; type I digenic lines: *1Dx5*+*1Dy10* and *NGli-D2* pyramids; type II digenic lines: *1Dx5*+*1Dy10* and *Sec-1^S^* pyramids; and trigenic lines: *1Dx5*+*1Dy10*, *NGli-D2* and *Sec-1^S^* pyramids. UPP%: ratio of unextractable polymeric protein to total glutenin polymeric protein.

**Table 4:** Effects of different gene pyramiding processes on agronomic traits.

Background	Pyramid	Spike Length (cm)	Spikelet Number	Grain Number per Spike	Plant Height (cm)	Thousand-Kernel Weight (g)
Hengguan 35	CK	9.56 ± 0.50a	16.60 ± 1.14a	50.60 ± 4.51a	82.20 ± 5.54a	42.86 ± 0.67b
	Type I digenic lines	9.94 ± 0.56a	17.00 ± 1.87a	51.20 ± 5.17a	76.40 ± 10.33ab	45.68 ± 4.23ab
	Type II digenic lines	9.72 ± 0.52a	15.60 ± 2.30a	54.20 ± 1.48a	71.20 ± 3.77b	48.36 ± 1.33a
	Trigenic lines	9.82 ± 0.86a	16.60 ± 1.87a	53.20 ± 5.26a	78.80 ± 4.60ab	42.10 ± 2.60b
Zhengmai 7698	CK	9.52 ± 0.47a	19.20 ± 2.39a	40.40 ± 1.67a	77.80 ± 3.27a	44.88 ± 0.32a
	Type II digenic lines	10.04 ± 0.30ab	21.40 ± 3.21a	45.40 ± 4.39ab	76.60 ± 5.27a	50.80 ± 7.96a
	Trigenic lines	10.26 ± 0.48a	21.00 ± 3.67a	47.20 ± 4.76a	72.60 ± 5.59a	45.58 ± 1.54a
Zhengmai 366	CK	9.92 ± 0.30a	19.40 ± 2.70a	39.60 ± 5.94a	69.60 ± 3.05a	42.42 ± 1.72b
	Type I digenic lines	10.04 ± 0.55a	21.00 ± 1.87a	43.60 ± 6.43a	72.20 ± 4.32a	46.34 ± 1.77a

The different lowercase letters in the table indicate significant differences between wild type and the pyramids according to Tukey’s test (*p* < 0.05). CK: control (wild type), type I digenic lines: *1Dx5*+*1Dy10* and *NGli-D2* pyramids, type II digenic lines: *1Dx5*+*1Dy10* and *Sec-1^S^* pyramids, and trigenic lines: *1Dx5*+*1Dy10*, *NGli-D2* and *Sec-1^S^* pyramids. Three years of data on BC_3_F_6_ to BC_3_F_8_ pyramids from 2019 to 2021 are used for agronomic trait analysis.

## Data Availability

Not applicable.

## Supplementary Materials

The following supporting information can be downloaded at: https://www.mdpi.com/article/10.3390/ijms24119253/s1.
