# Supplementary material for: Quality and Agronomic Trait Analyses of Pyramids Composed of Wheat Genes NGli-D2, Sec-1s and 1Dx5+1Dy10
Source: Int J Mol Sci. 2023 May 25;24(11):9253. doi: 10.3390/ijms24119253 (PMC10252852; doi:10.3390/ijms24119253)
Supplement: Supplementary file 1 [file ijms-24-09253-s001.zip › ijms-2389008-supplementary.pdf]

Table S1 Results of polymeric identification of genes from different genetic backgrounds

Parent	Generations	Gene polymerization types	Number of tested plants	Number of polymers	Polymerization rate
Hengguan35	F_3	*1Dx5+1Dy10*、*Sec-1^S^*	96	6	6.3%
		1Dx5+1Dy10、*NGli-D2*、*Sec-1^S^*	48	1	2.1%
	F_4	*1Dx5+1Dy10*、*Sec-1^S^*	107	34	31.8%
		1Dx5+1Dy10、*NGli-D2*、*Sec-1^S^*	260	10	3.8%
	F_5	*1Dx5+1Dy10*、*Sec-1^S^*	200	101	50.5%
		1Dx5+1Dy10、*NGli-D2*、*Sec-1^S^*	120	10	8.3%
	F_6	*1Dx5+1Dy10*、*NGli-D2*	107	14	13.08%
		1Dx5+1Dy10、*Sec-1^S^*	113	75	66.37%
		1Dx5+1Dy10、*NGli-D2*、*Sec-1^S^*	26	12	46.15%
	F_7	*1Dx5+1Dy10*、*NGli-D2*	80	54	67.50%
		1Dx5+1Dy10、*Sec-1^S^*	139	133	95.68%
		1Dx5+1Dy10、*NGli-D2*、*Sec-1^S^*	50	11	22%
	F_8	*1Dx5+1Dy10*、*NGli-D2*	140	127	90.7%
		1Dx5+1Dy10、*Sec-1^S^*	136	131	96.3%
		1Dx5+1Dy10、*NGli-D2*、*Sec-1^S^*	60	49	81.7%
Zhengmai 7698	F_3	*1Dx5+1Dy10*、*Sec-1^S^*	86	10	11.6%
		1Dx5+1Dy10、*NGli-D2*、*Sec-1^S^*	35	1	2.8%
	F_4	*1Dx5+1Dy10*、*Sec-1^S^*	553	145	26.2%
		1Dx5+1Dy10、*NGli-D2*、*Sec-1^S^*	500	21	4.2%
	F_5	*1Dx5+1Dy10*、*Sec-1^S^*	240	111	46.3%
		1Dx5+1Dy10、*NGli-D2*、*Sec-1^S^*	150	15	10%
	F_6	*1Dx5+1Dy10*、*Sec-1^S^*	107	32	29.91%
		1Dx5+1Dy10、*NGli-D2*、*Sec-1^S^*	134	50	37.31%
	F_7	*1Dx5+1Dy10*、*Sec-1^S^*	366	260	71.04%
		1Dx5+1Dy10、*NGli-D2*、*Sec-1^S^*	89	84	94.38%
	F_8	*1Dx5+1Dy10*、*Sec-1^S^*	176	163	92.6%

		1Dx5+1Dy10、*NGli-D2*、*Sec-1*[S]	208	175	84.1%
Zhengmai 366	F_3	*1Dx5+1Dy10*、*NGli-D2*	129	16	12.4%
	F_4		463	56	12%
	F_5		450	55	12.2%
	F_6		565	66	11.68%
	F_7		161	155	96.27%
	F_8		216	179	82.9%
